# Designing of Green Plasticizers and Assessment of the Effectiveness of Their Use

**DOI:** 10.3390/polym13111761

**Published:** 2021-05-27

**Authors:** Aliya K. Mazitova, Guliya K. Aminova, Irina N. Vikhareva

**Affiliations:** Applied and Natural Sciences Department, Ufa State Petroleum Technological University, Mendeleeva St. 195, 450080 Ufa, Russia; elenaasf@yandex.ru (A.K.M.); aminovagk@inbox.ru (G.K.A.)

**Keywords:** adipate plasticizer, biodegradable, economic efficiency, environmental damage, esterification, polyvinyl chloride

## Abstract

The growing anthropogenic load on the lithosphere is currently characterized by the alienation of huge areas for solid domestic waste. One of the most common pollutants is traditional plastics with a degradation period of over 100 years. In connection with the increasing environmental requirements, polymer materials, along with a high set of technological and operational parameters, must be environmentally friendly and biodegradable. The development of polymer composite materials that undergo accelerated physicochemical and biological changes in the natural environment due to the introduction of biodegradable additives is one of the potential methods for processing synthetic materials and ensures the release of significant areas of fertile soils and lands from the steadily increasing amount of polymer waste. The use of adipic acid esters as PVC plasticizers contributes to the production of biodegradable composites. The article describes a method for obtaining new esters of adipic acid, presents the results of studying their properties for practical use in PVC composites, and assesses the economic efficiency of preventing damage to the environment when using them.

## 1. Introduction

Environmental pollution as a result of human activities leads to a deterioration in the health indicators of the population. In modern conditions, the provision of chemical safety is one of the priority tasks of socio-economic development [1,2,3,4]. Polymeric materials, as the most popular and widespread, make a significant contribution to the deterioration of the ecological situation [5,6,7,8]. The disposal of polymer products after operation most often occurs at solid waste landfills or is spontaneous.

Soil pollution leads to degradation of the soil cover, withdrawal of land from agricultural use, and significant financial costs for the implementation of measures for the remediation of contaminated land [9,10,11,12]. In addition, soil contamination with waste polymer materials poses a significant danger due to the migration ability of some toxic additives [13,14].

Soil is the first link in the food chain, and the quality of plant and animal products, as well as secondary pollution of atmospheric air and water resources, largely depend on its properties. The process of accumulation of pollutants in the soil is much slower than in other environmental objects, but at the same time, pollutants in the soil persist for a long time, which subsequently leads to secondary pollution of atmospheric air, surface and groundwater, disruption of self-purification processes in the soil, and its deterioration. The fertility and growing conditions of plants have a negative impact on the health of the population [15,16,17]. In addition, the waste of polymeric materials, when disposed of in the soil, can be transformed into more toxic compounds, adsorbed and accumulated. Soil contaminated with persistent chemicals is a source of pollution of the environments in contact with it and then through biological chains, entering the human body, has an adverse effect on health [18].

Therefore, at present, the problem of massive soil pollution as a universal sorbent and accumulation medium is of great ecological and economic importance [19,20,21].

The prevention of environmental damage, as expressed in lithosphere pollution with solid household waste and waste products from polymeric materials generated at solid waste landfills, directly contributes to the economic effect of the development and introduction into the production of biodegradable polymer composite materials [22,23].

In this regard, it is extremely important to produce polymer composite materials with a controlled service life, which retain their operational characteristics during the period of their use, and then degrade under environmental conditions with the formation of non-toxic substances. One of the promising ways to create such materials is the development of composite materials based on synthetic polymers with additives that ensure the decomposition of the entire composition [24,25,26].

In recent years, biodegradable polymers from renewable sources have attracted a lot of research attention. The main reasons contributing to the development of this direction include the ability to complete biodegradation in the environment, reduction of hydrocarbon reserves, reduction of waste and compostability in the natural cycle, and climate protection by reducing the amount of carbon dioxide emitted [27,28].

It is expedient to modify polymer compositions using plasticizers capable of serving as a source of organic substances for microorganisms destructors under ambient conditions.

The use of natural biodegradable plasticizers with low toxicity and good compatibility with traditional polymers is a growing trend in the development of bioplastics. Epoxidized triglyceride vegetable oils from soybean oil, linseed oil, castor oil, sunflower oil, and fatty acid esters are widely used as natural-based plasticizers [29,30,31,32]. However, in some applications, a complete substitution with natural-based plasticizers is simply not possible, which is why a wide range of commercially available biodegradable synthetic plasticizers have been developed over the past decades: esters of adipic, azelaic, sebacic, citric, and tartaric acids [33].

Thus, expanding the range of biodegradable plasticizer additives based on adipic acid esters is a topical effective way to increase the biodegradation of PVC composites. 

## 2. Materials and Methods

### 2.1. Starting Materials

Adipic acid (purity 99.8%) used in the synthesis of esters was purchased from Radici Group, Selbitz—Hochfranken, Bavaria, Germany. Butanol (purity 99.7%) and Decanol (purity 99 %) were purchased from The Company «Rearus», Moskow, Russia. Phenol was used as received from “Ufaorgsintez,” Ufa, Russia. Ethylene oxide (ECOTECH Chemical Components Plant, Moscow, Russia) is a liquefied gas that is a colorless transparent liquid in steel cylinders with a boiling point (m.b.) of 10.7 °C. Sodium hydroxide used as catalyst, was of 98.2%, and it was purchased from Joint Stock Company “Caustic,” Sterlitamak, Russia. P-Toluenesulfonic acid used as catalyst (purity 95 %), it was purchased from Component-Reagent, Moscow, Russia. O-Xylene (purity 99%) was purchased from Public Joint-Stock Company “Joint-Stock Oil Company Bashneft,” Ufa, Russia. Polyvinyl chloride (Joint Stock Company “Caustic,” Sterlitamak, Russia): We used industrial samples of suspension polyvinyl chloride PVC 7059M. Suspension polyvinyl chloride made by suspension polymerization, with a K value from 70 to 73, with a bulk density from 0.45 to 0.55 g/cm^3^, with a residue after sieving on a sieve with a mesh N 0063–95%, for the manufacture of plasticized products. It is a homogeneous white powder.

### 2.2. Synthesis Methods

#### 2.2.1. Synthesis of Ethoxylated Alcohol

Ethoxylated alcohols were prepared as described previously [30]. Briefly, the calculated amount of alcohol and sodium hydroxide catalyst in the flask is heated to 110–120 °C and then purged with nitrogen to remove air; then, prepared ethylene oxide is gradually introduced. The required temperature of the reaction mixture is maintained within the specified interval for 1 h and then cooled to room temperature. The catalyst is neutralized, the resulting mass is filtered, and the light fraction is distilled off from the reaction mixture, boiling off to 50 °C at 10 mm Hg.

Butoxyethanol is a colorless oily liquid soluble in water. The yield was 106.8 g (90.5% of theoretical).

Phenoxyethanol is a colorless oily liquid soluble in water. The yield was 122.8 g (89% of theoretical).

#### 2.2.2. The Synthesis of Unsymmetrical Esters of Adipic Acid

Unsymmetrical adipates were obtained according to the method described earlier [30]. Briefly, 150 mL of o-xylene, 1 mol of ethoxylated alcohol, 3.0 g (1% by weight) of p-toluenesulfonic acid, and 146 g (1 mol) of adipic acid were charged into the reaction flask, and the temperature was slowly raised to 90–100 °C. Stirring was continued for 1.5 h. The end of the reaction was determined by the acid number of the esterificate.

Then, it was esterified with aliphatic alcohol (1.2 mol). Boiling was continued for 2–3 h. The reaction mixture was cooled and the target ester was isolated. 

Decyl butoxyethyl adipate is the light clear liquid. The yield was 343.5 g (89.0% of theoretical).

Decyl phenoxyethyl adipate is the light powder product. The yield was 365 g (90.0% of theoretical).

### 2.3. Methods of Analysis

The study of the composition of the reaction masses formed during the synthesis of esters of adipic acid was carried out by high-performance liquid chromatography (LC-10 from SHIMADZU, Tokyo, Japan) in a reversed-phase mode. The separation of the mixture components was carried out on a column (150 × 4.6 mm) filled with a Separon-C18 sorbent with a particle size of 5 μm in an acetonitrile–water eluent system, taken in a 67/33 volumetric ratio. The eluent flow rate is 0.5 mL/min. A refractometric model detector (RIDK 101, Czech Republic, Prague) was used as a detector. The volume of injected samples was 10 μL. Quantitative analysis was performed using the absolute calibration method. The calibration solutions contained adipic acid and alcohols.

#### 2.3.1. Analysis of Physicochemical Parameters of Plasticizer

The analysis of physicochemical parameters of the obtained compound was carried out in accordance with state standard 8728-88 “Plasticizers: Specifications” [34]. For this, the following indicators were determined: acid number, ester number, mass fraction of volatile substances.

Determination of plasticizer density according to state standard 18329–2014 “Liquid resins and plasticizers: Methods for determination of density” [35].

#### 2.3.2. Characterization of Esters of Adipic Acid

Samples of the obtained products were analyzed by FTIR spectroscopy (KBr tablets), which were prepared according to a standard procedure. IR absorption spectra were recorded in the range 450–3700 cm^–1^ using an FTIR-8400S FTIR spectrometer (Shimadzu, Tokio, Japan) at room temperature. Resolution—4 cm^–1^, number of scans—20. 

#### 2.3.3. Determination of Thermal Stability of Obtained Plasticizer

Determination of thermal stability of plasticizer and polymeric compositions based on polyvinyl chloride. To determine the thermal stability of plasticizer and polymer compositions based on polyvinyl chloride, a TGA/DSC-1 thermal analysis device (Mettler Toledo, Greifensee, Switzerland) was used. The following paragraphs outline the effects accompanying the sample heating process. 

The measurements of the thermal stability of the sample were carried out in the temperature range from 25 to 500 °C in air. The measurements were carried out in a dynamic mode with a constant heating rate of 5 K/min. The mass of the sample used for measurements was 5–10 mg. For the analysis, we used alumina crucibles with a volume of 100 μL. The cooling rate of the device is 20 K/min. The measurement error is ±0.3 K. The results were processed using a computer and STARe thermal analysis software.

When conducting research on the TGA-DSC-1 thermal analyzer, the following curves are recorded on the resulting thermogram of the sample, which were used to assess the thermal stability of the samples:

- Thermogravimetric curve (TG) characterizes the change in the sample mass during heating (the dependence of the mass on temperature);
- Differential thermogravimetric curve DTG characterizes the rate of change in the sample mass during heating;
- The DSC curve characterizes the thermal effects observed during the heating of the sample.

**Determination of melting and crystallization temperatures of plasticizer.** The melting and crystallization points of the plasticizer were determined by differential scanning calorimetry on a DSC-1 instrument. The temperature range of the device is from −150 to 500 °C. The sample is cooled with liquid nitrogen. The sample heating rate varies in the range from 0.02 to 300 K/min. The cooling rate of the device is from 0.02 to 50 K/min. The measurement error ±0.2 K.

The measurements were carried out in the temperature range from −50 to 150 °C in air. The measurements were carried out in a dynamic mode with a constant sample heating/cooling rate of 10 K/min. The mass of the sample used for measurements was 4–8 mg. For the analysis, aluminum crucibles with a volume of 40 μL were used. A weighed sample was placed in a crucible and sealed with a lid using a press.

The melting point of the plasticizer was determined from the DSC curve obtained in the sample heating mode. The melting point of the plasticizer corresponds to the maximum value of the endothermic peak observed on the DSC curve.

The crystallization temperature of the plasticizer was determined according to the DSC curve obtained in the cooling mode of the sample after its preheating by 30–40 °C higher than its melting point. The crystallization temperature of the plasticizer corresponds to the maximum value of the exothermic peak observed on the DSC curve.

**Determination of the glass transition temperatures of PVC compositions.** The glass transition temperatures of PVC composites were determined by differential scanning calorimetry on a DSC-1 instrument (Mettler Toledo, Greifensee, Switzerland).

The measurements were carried out in the temperature range from −20 to 150 °C in air. The measurements were carried out in a dynamic mode with a constant heating rate of 10 deg/min. The mass of the sample used for measurements was 4–8 mg. For the analysis, aluminum crucibles with a volume of 40 μL were used. A weighed sample was placed in a crucible and sealed with a lid using a press.

The glass transition temperature of the polymer was determined from the DSC curve obtained in the heating mode of the sample. Using the tangent method, the middle of the bend (step) on the curve was determined, which was taken as the glass transition temperature.

#### 2.3.4. Determination of Glass Transition Temperature of Obtained Plasticizers

Glass transition temperatures of PVC composites were determined by differential scanning calorimetry on a DSC-1 instrument (Mettler Toledo, Greifensee, Switzerland).

The analysis was carried out in the temperature range from −100 to 100 °C in atmosphere air. The measurements were carried out in a dynamic mode at a constant heating rate of 2 deg/min. The mass of the sample taken for measurements was 4–8 mg. For the analysis, aluminum crucibles with a volume of 40 μL were used. A weighed sample was placed in a crucible and sealed with a lid using a press. After quenching at 90 °C for 5 min, the sample was heated to 100 °C at a rate of 2 °C/min. The first heating cycle was used to remove any heat history. The glass transition temperature of the polymer was determined from the DSC curve obtained in the second heating cycle of the sample using the supplied software. The tangent method was used to determine the middle of the bend (step) on the curve, which was taken as the glass transition temperature.

#### 2.3.5. Determination of Fungal Resistance of Samples of PVC Films

Determination of fungal resistance of samples was carried out according to state standard 9.049–91 (ISO 846-78) “Unified system of corrosion and aging protection: Polymer materials and their components. Methods of laboratory tests for mould resistance”. For this, three species of mycelial fungi were used, which are the main biodestructors of various materials, including polymeric ones: *Aspergillus niger*, *Pénicillium sp*., and *Paecilomyces sp*. A suspension of fungi spores with a spores concentration of each fungus species of 1–2 ppm/cm^3^ was used.

Samples of PVC films of size 3.0 × 3.0 cm and thickness 1.0 ± 0.2 mm were purified from external contaminants by immersion for 1 min in ethanol and then dried. The samples thus prepared were placed in sterile petri dishes.

For the study, the films were infected with mold fungi spores in a solution of mineral salts with sugar (Chapek–Doks medium): The surface of the samples was uniformly sprayed with a suspension of fungi spores, preventing the droplets from draining, and then kept in the box until the droplets dried; the PVC film surface was treated on the other side in a similar way.

Test samples of films in glass Petri dishes were kept under optimal conditions for the development of fungi: in a thermostat at a temperature of 30 °C for 28 days with intermediate inspection after 7 and 14 days.

After 5 days, control Petri dishes were examined for the viability of fungal spores. Subsequently, after every 7 days, the exciter cover was opened for 3 min to inflow air.

During the intermediate examinations and at the end of the tests, samples were removed from the chamber or exicator, examined with the naked eye in scattered light at an illumination of 200–300 lx and at an increase of 56–60 times. Antifungal resistance is assessed by mushroom development intensity on samples on a 6-point scale. Samples without treatment with microscopic fungi acted as controls.

Statistical processing of the obtained data was carried out using the computer program Excel 2007, calculating the average arithmetic and standard error of the average. The validity of differences between mean values was evaluated by Student’s t-test for the significance level a = 0.05.

#### 2.3.6. Definition of Indicators of Tensile Stress and Elongation at Break of Samples of PVC Films

The definition of indicators is described in the work [29], which was carried out in accordance with the state standard 9998–86 [36]. 

#### 2.3.7. Study of the Biodegradation of PVC Films in Compost

The study of the biodegradation of polymer composite films was carried out by the composting method described in state standard 9.060—“Fabrics: Method of laboratory tests for microbiological destruction stability” [37]. During the experiment, the model compost consisted of a mixture of soil, manure, and sand in a ratio of 1:1:1. Before testing in soil, the pH of the aqueous extract is determined. The soil is considered suitable for testing at a pH of 6.0–7.5. Samples of composites 150 × 150 mm in size were immersed in compost and kept in this environment for 10, 30, 45, 90, and 120 days at a temperature of 28 °C and a relative humidity of 65%. After aging, the films were washed from the compost with distilled water and dried at a temperature of 60 °C for 24 h.

Determination of the pH of the water extract of the soil. First, 10 g of soil was crushed in a mortar and sifted through a sieve, which was followed by pouring 25 cm^3^ of distilled water. After 30 min with constant stirring and at room temperature, the pH was measured. Three parallel measurements were made, and the average value was taken with an error of no more than 0.1.

### 2.4. Preparation of Film Samples

The following composition was prepared for testing: 100 parts by weight of PVC; 42 parts by weight of the plasticizer; 3 parts by weight of calcium stearate stabilizer.

To obtain test samples, all the ingredients of the PVC composition were mixed in a two-stage laboratory mixer for 60 min. To study the PVC composition, samples were obtained in the form of rigid and plasticized films. PVC film samples were obtained by rolling on laboratory rollers at temperatures of 165–175 °C for 5 min.

## 3. Results

The oxyethylation reaction of alcohols has been well studied [38,39,40]. The ethoxylation of alcohols and the characteristics of the products are described in our previous works [25,30]. 

**Scheme 1:**
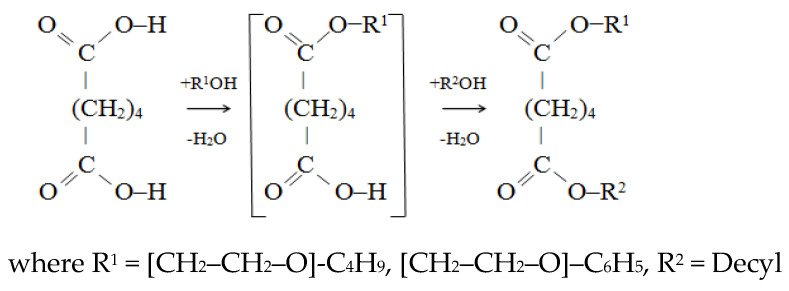
Synthesis of unsymmetrical esters of adipic acid.

The synthesis of unsymmetrical adipates of ethoxylated alcohols was carried out by a two-stage esterification of adipic acid in one reaction volume (Scheme 1). At the first stage, at an equimolar ratio of the starting reagents, a monoester of adipic acid was obtained.

Without isolating the latter, pre-esterification was carried out with an excess of the corresponding alcohol at the boiling point of the reaction mixture. To facilitate the removal of the evolved water, the synthesis was carried out in the medium of an azetropic water carrier o-xylene, and the reaction mass was bubbled with an inert gas. During the synthesis, the acid number of the reaction mixture was monitored. The flask contents were cooled. Furthermore, the isolation of the target ester was carried out depending on the aggregate state of the product. The obtained decyl butoxyethyl adipate is oily yellowish liquid, and decyl phenoxyethyl adipate is light powdery product. Target esters were obtained with a yield of at least 80%.

The physicochemical properties of the obtained adipates are presented in Table 1.

**Figure 1:**
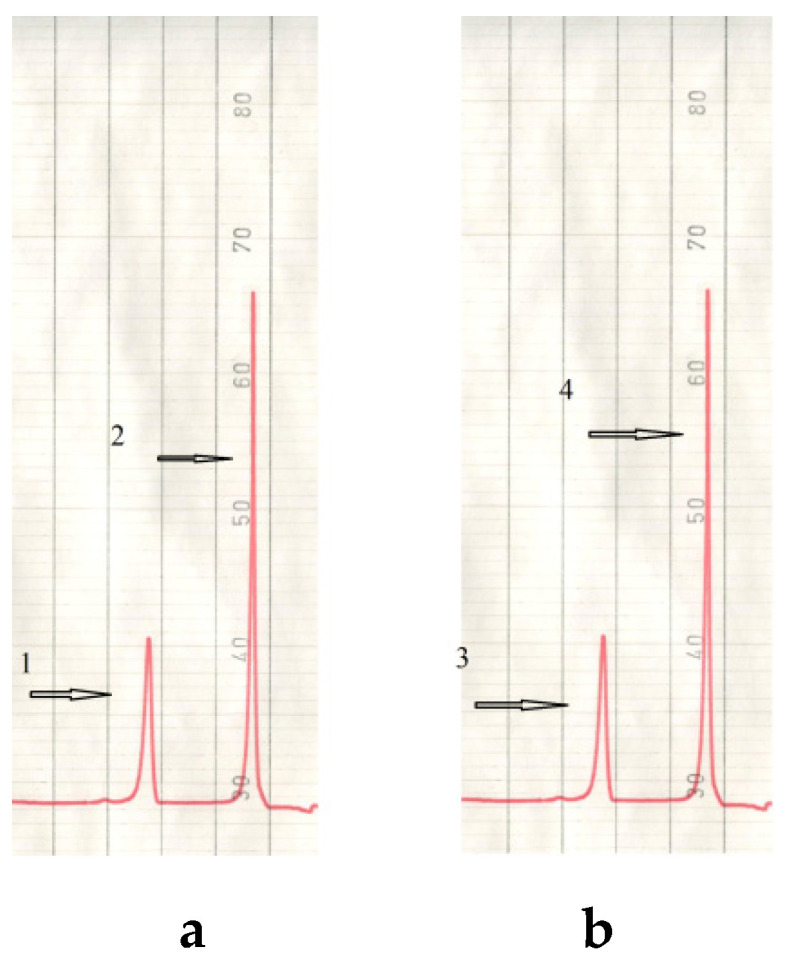
Typical chromatogram of adipic acid esters. (**a**) 1—dibutoxyethyl adipate, 2—decyl butoxyethyl adipate; (**b**) 3—diphenoxyethyl adipate, 4—decyl phenoxyethyl adipate.

HPLC analysis was performed to check the chemical composition of the resulting product. In both cases, the obtained chromatograms clearly show two products: in the first case, decyl butoxyethyl adipate and dibutoxyethyl adipate; in the second case, decyl phenoxyethyl adipate and diphenoxyethyl adipate (Figure 1). The method of carrying out the synthesis suggests that in each case of esterification, the product may contain three esters: an unsymmetrical ester and two symmetric esters. At the first stage, during the interaction of adipic acid and decanol, the formation of the monoester, didecyladipinate is possible, and, probably, some amount of adipic acid will remain unreacted. Further, in the second stage, with the addition of a lower molecular weight alcohol (butoxyethanol or phenoxyethanol), a corresponding unsymmetrical ester and a small amount of a symmetric ester of dibutoxyethyl adipate or diphenoxyethyl adipate are formed. The didecyl adipate formed in the first stage undergoes transesterification with a lower molecular weight alcohol. The purity of the reaction products obtained is quite satisfactory, since plasticizers are technical products and allow the presence of impurities up to 5%.

**Figure 2:**
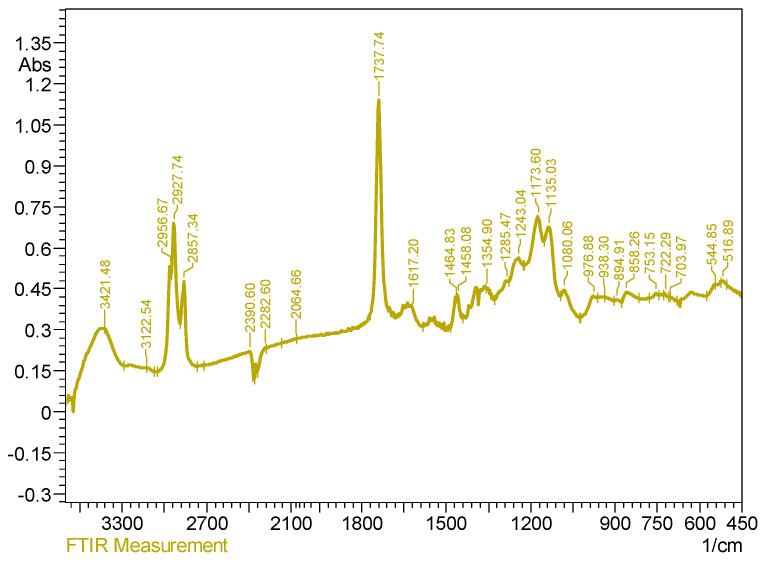
FTIR spectra of decyl butoxyethyl adipate.

**Figure 3:**
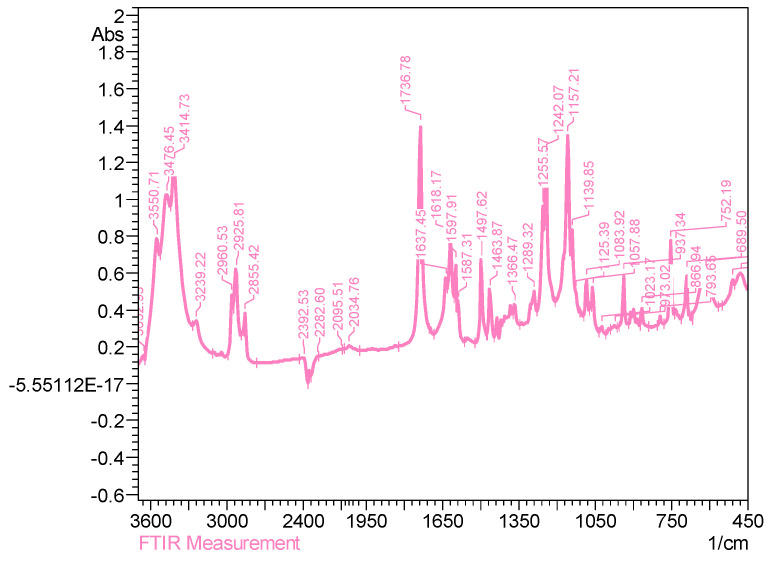
FTIR spectra of decyl phenoxyethyl adipate.

The preparation of esters of adipic acid was confirmed by IR spectra (Figure 2 and Figure 3). Thus, the spectra of the synthesized adipates lack an absorption band in the range of 1685–1687 cm^−1^, which is characteristic of stretching vibrations of the carbonyl group in associates of aliphatic carboxylic acids. Unlike carboxylic acids, in esters, the stretching vibrations of the carbonyl group are shifted to the high-frequency region and appear as a strong characteristic band in the 1750–1735 cm^−1^ range. The IR spectra of the obtained products exhibit absorption bands corresponding to the ester bands: in the region of 1737 cm^−1^ for DBEA and 1736 cm^−1^ for DPEA, which correspond to stretching vibrations of the carbonyl group in esters, and absorption bands at 1157 cm^−1^ for decyl butoxyethyl adipate and 1173 cm^−1^ for decyl phenoxyethyl adipate, which correspond to the vibrations of the C–O–C ester fragment.

In addition, in the IR spectra of esters, in contrast to acids, there is no very broad (“acidic”) band in the range of 3500–2500 cm^−1^ with a number of small peaks, and the band of bending vibrations of the CH_3_ group at about 1360 cm^−1^ is clearly manifested. In acids, this band is very weak. Stretching vibrations of the C–O group in the IR spectra of esters, as well as in the spectra of acids and alcohols, give a number of strong bands in the range of 1300–1000 cm^−1^, but they are not characteristic. Nevertheless, it is possible to distinguish the average vibration frequencies ν(C–O) for a number of esters with a hydrocarbon chain. The strongest peak is observed at about 1139 cm^−1^ for DPEA and 1157 cm^−1^ for DBEA. In addition to it, esters of such acids have two more weaker bands associated with C–O vibrations: peaks at about 1243 cm^−1^ and 1285 cm^−1^ for DBEA; about 1255 cm^−1^ and 1242 cm^−1^ for DPEA.

The presence of an aromatic substituent in the DPEA ether manifests itself in the IR spectra as peaks at 1597 cm^−1^ and 1497 cm^−1^, corresponding to the vibrations of the C–C bond.

Then, for the practical application of asymmetric esters of adipic acid as plasticizers of PVC composites, the thermoanalytical parameters and compatibility of the obtained products with PVC were determined.

**Figure 4:**
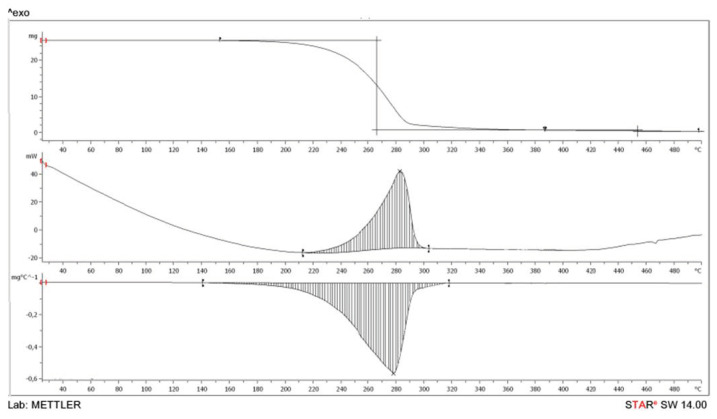
TGA–thermogram of the decyl butoxyethyl adipate.

**Figure 5:**
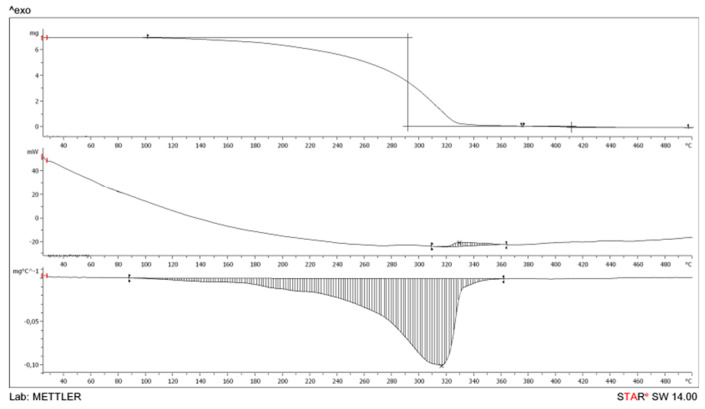
TGA–thermogram of the decyl phenoxyethyl adipate.

The determination of thermoanalytical parameters of plasticizers was carried out on a TGA-DSC device for combined thermal analysis (Mettler Toledo) in the temperature range 20–500 °C at a heating rate of 5 °C/min. (Figure 4 and Figure 5). The industrial plasticizer di-2-ethylhexyl phthalate (DOP) was used as a reference.

An important indicator for the use of a plasticizer during processing as part of a PVC composite is the change in the weight of the additive when heated in a similar temperature regime. The results of heating the plasticizer to a temperature of 180 °C are presented in the Table 2.

On the obtained TGA thermograms, the decomposition of the developed plasticizers occurs in the temperature range from 101 to 499 °C (Table 2). The plasticizer decyl butoxyethyl adipate is characterized by the highest temperature value corresponding to the beginning of the decrease in the weight of the plasticizer upon heating, and it exceeds the industrial DOP by 21 °C (Table 2).

The values of another parameter, the temperature corresponding to the maximum decomposition rate of the product, for the studied plasticizers are in a fairly wide temperature range from 278 to 316 °C. The introduction of a phenoxyethyl fragment (DPEA) into the ester molecule improves this indicator (316 °C, respectively).

From the results of thermogravimetric analysis, it follows that the plasticizer decyl phenoxyethyl adipate has a higher thermal stability among the studied products, which surpasses the industrial plasticizer DOP in this parameter, which ensures good processability of PVC compounds based on it.

Relatively low values of the parameter Δm of the additive at a temperature of 180 °C, which characterizes the content of volatile impurities in the additive, which can migrate during the formation of materials and products, are important for the use of the developed esters as PVC plasticizers. These indicators for all studied compounds were lower in comparison with industrial DOP (Table 2).

The DTG thermogram of the obtained esters shows the boundaries corresponding to the change (decrease) in the sample weight upon heating. The peak area can be used to judge the intensity of the decomposition process. The maximum of the peak corresponds to the maximum rate of weight loss (decomposition) of the sample. It can also be observed from the DTG curves that the additives obtained exhibit a one-step degradation behavior.

However, the synthesized DPEA is a solid product, so the melting and crystallization temperatures are important indicators for its practical use.

On DSC thermograms obtained by heating the samples, the butoxyethyl derivative of adipic acid is characterized by a melting point—(−40.3) °C, and the value of the enthalpy of fusion is −14.1 J/g (Table 3), while the phenoxyethyl derivative of adipic acid is characterized by a melting point −84.0 °C, and an enthalpy of fusion is −67.8 J/g (Table 3).

The DSC thermograms obtained upon subsequent cooling of the samples clearly show exothermic peaks corresponding to the crystallization of plasticizers: butoxyethyl derivative of adipic acid is characterized by a crystallization temperature of −60.5 °C and the value of the enthalpy of crystallization is 9.4 J/g (Table 3), the phenoxyethyl derivative of adipic acid is characterized by a crystallization temperature of −8.0 °C and the value of the crystallization enthalpy is 131.2 J/g (Table 3).

**Figure 6:**
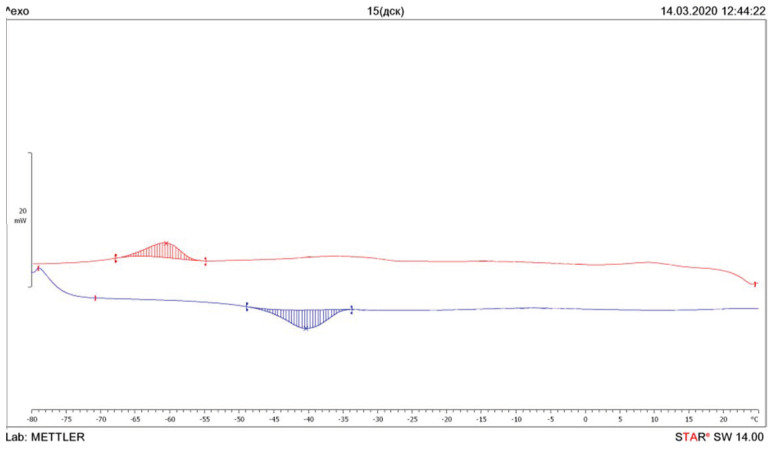
DSC thermogram of the decyl butoxyethyl adipate.

**Figure 7:**
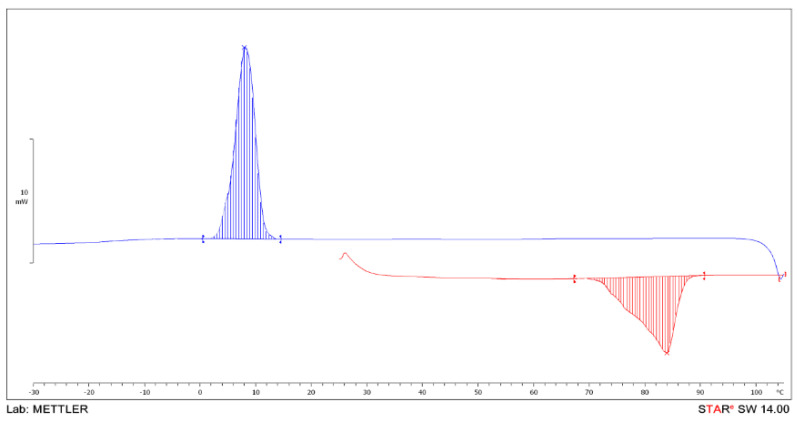
DSC thermogram of the decyl phenoxyethyl adipate.

There is a noticeable difference in temperatures and enthalpies of melting and crystallization of products for several reasons (Figure 6 and Figure 7). First, the crystallization process is always shifted relative to melting to the region of lower temperatures. Secondly, at a cooling rate of 10 deg/min, the crystallization process may be incomplete. In addition, several other factors influence the melting and crystallization processes. 

Thus, the thermoanalytical studies carried out have shown that the developed adipate plasticizers have a higher thermal stability in comparison with DOP: On the TGA thermograms, shifts of the exothermic peaks of thermooxidative decomposition toward higher temperatures are observed. DSC thermograms of the obtained plasticizers show that in the course of processing, these additives in PVC compositions improve the manufacturability of the compounds.

The compatibility of PVC with plasticizers is one of the most important factors that determines the suitability of their use in the development of the composition of the plasticized material.

The process of diffusion of a plasticizer from a place with a higher concentration to a place with a lower concentration is the migration of the plasticizer [41]. To a large extent, the magnitude of migration is determined by the nature of the polymer that interacts with the plasticizer.

The lower the limit of compatibility of the plasticizer with the polymer, the less their interaction, and the higher the amount of migration [42]. 

One of the ways to determine the compatibility of a plasticizer with PVC is to estimate the critical temperature of dissolution of the polymer in the plasticizer. Tests have shown that the dissolution temperature of PVC in the developed plasticizers is higher than in DOP (Table 4). Moreover, for decyl phenoxyethyl adipate, this indicator is significantly higher than for decyl butoxyethyl adipate, which indicates a lower compatibility with the polymer. Probably, the obtained unsymmetrical ester of adipic acid and phenoxyethanol can be attributed to secondary plasticizers, which are partially compatible with the polymer and are used as a mixture with a primary plasticizer, for example, with DOP.

A decrease in the glass transition temperature of a polymer upon the introduction of a plasticizer is an important criterion for assessing the effectiveness of its plasticizing action.

The glass transition temperature was determined by DSC on a DSC-1 instrument (Mettler Toledo) at a heating rate of 2 deg/min. The transition of a polymer from a glassy to a highly elastic state is accompanied by an increase in the heat capacity of the polymer, which is reflected in the form of a characteristic break (step) on the DSC curve. The Tg value was found in the middle of the step corresponding to this transition.

**Figure 8:**
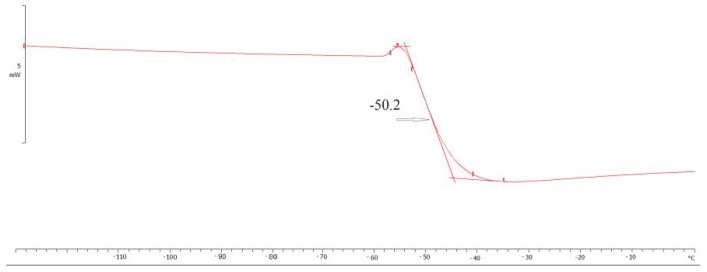
DSC thermogram of PVC composition containing DBEA.

**Figure 9:**
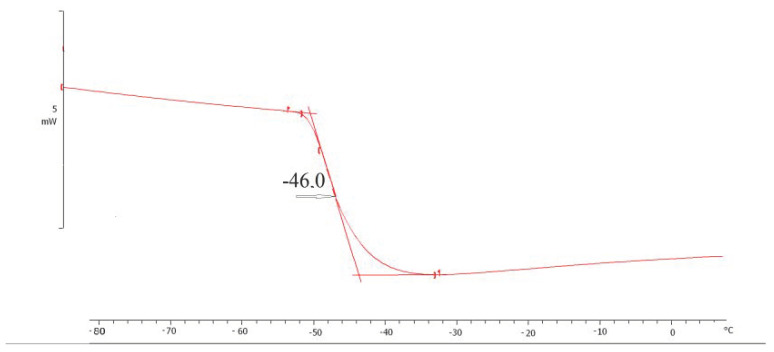
DSC thermogram of PVC composition containing DPEA.

To assess the effect of plasticizers on Tg, plasticized PVC compositions with a plasticizer content of 50 parts by weight were used for 100 parts by weight of PVC (Figure 8 and Figure 9).

Thus, the introduction of the products decyl butoxyethyl adipate (Tg = −50.2) and decyl phenoxyethyl adipate (Tg = −46.0) into the PVC composition leads to a noticeable decrease in the glass transition temperature of the polymer, which indicates a high efficiency of the plasticizing action of these compounds.

To study the effect of the obtained plasticizers on the biodegradation of PVC plastics, PVC films of the following composition were obtained: 100 parts by weight of PVC; 40 parts by weight of the plasticizer; 3 parts by weight of calcium stearate stabilizer.

To assess the biostability, the obtained film samples were tested by a mycological test. It was found that in the first week of incubation on a sample of composition 1, there is an initial growth of mold cultures with the formation of foci of mycelium and the formation of sporulation. Further, the active growth of micromycete colonies occurs, as a result of which the material of film of composition 1 becomes moldy, which indicates the presence of an available substrate in the system and confirms the bioavailability of the developed additives in the composition of PVC composites for various genera of microscopic fungi. The fouling of sample of composition 1 is observed already on the 14th day of infection, and the process of fungal growth continues throughout the experiment.

When testing the sample of composition 2 for 28 days, less growth of moldy cultures with the formation of foci of mycelium is observed, which is probably due to the presence of a phenoxyethyl group in the molecule of the corresponding adipate.

The assessment of the biostability of the developed composites was carried out on the 28th day of the experiment (Table 5). The sample numbers in Table 4 correspond to the composition numbers in Table 5.

At the end of the incubation period, it was found that polymer composition 1 to 2 can be used as a food source for microscopic fungi such as Aspergillus niger, Pénicillium funiculosum, and Trichoderma lignorum. Hence, the materials contain enough nutrients to support the growth of the fungi.

Plasticizers in PVC-based materials have different resistance to microorganisms. The nature of the plasticizer plays an important role in this. When microorganisms use plasticizers as a carbon source in PVC materials, significant changes in properties are observed.

It is known that unlike phthalate plasticizers, adipate additives are involved in the life of various microorganisms, resulting in the formation of acidic products soluble in water. For example, oxalic and succinic acids, which accelerate the decomposition of the material [43,44]. Furthermore, weakening of the polymer structure leads to changes in the molecular weight and mechanical properties of plastic compounds. Residues of polymer molecules are perceived by microorganisms as nutrients, which leads to an increase in their population.

Thus, the conducted mycological test showed the possibility of accelerated biodegradation of PVC compounds based on the developed adipate plasticizers under environmental conditions.

The most suitable biological environment for the degradation of polymer products is soil, since it contains a variety of microorganisms, such as soil bacteria, fungi, etc., that contribute to the decomposition process.

The study of the effect of composting on the performance characteristics of PVC films after the end of their service life is one of the important tasks associated with the recycling of polymer waste.

In the course of the work, a study was carried out on the composting of PVC composite films for 120 days. The results obtained are presented in Table 6 and Table 7.

An analysis of the results obtained (Table 6 and Table 7) showed an increase in tensile breaking stress and a corresponding decrease in the elongation at break, which indicates a decrease in the elasticity of the films as a result of destruction and loss of the plasticizer. The observed change in the physicomechanical characteristics for composition 2 was somewhat lower, which is probably due to the presence of a phenoxyethyl group in the molecule of the corresponding plasticizer included in the PVC composite formulation.

The biodegradation of hybrid polymer composites based on large-tonnage synthetic polymers and biodegradable additives under the influence of soil microorganisms consists in the repeated passage of processes: surface biocorrosion, the formation of a more porous structure (due to washout of fillers), internal biocorrosion (due to fixation of micromycetes on internal irregularities), the spread of erosion, fragmentation. As a result, polymeric materials, being organic compounds, can undergo biological degradation, i.e., they can be disposed of.

The ability to accelerate the biodegradation of the obtained PVC compounds is an indisputable advantage of the developed additives. The process of destruction of material when it enters the soil occurs under the action of microorganisms and is accompanied by a significant drop in its strength. The degradability of polymeric materials in nature depends on the structure of the polymer, the presence of a population of degrading microorganisms, and the environmental conditions that favor their growth. Under the influence of free radicals and various microorganisms, the resulting fragments are involved in hydrolytic and redox processes, which leads to a decrease in molecular weight and further simplifies and accelerates the process of biodegradation of polymer plastic. 

However, in the literature, there are many studies on photodegradation and thermal decomposition of PVC, but there are only a few reports on its biodegradation. The degradation process of polymers is still poorly understood, and there is especially little information about the mechanisms involved and the microorganisms involved.

The existence of organisms capable of metabolizing synthetic polymers has been of considerable interest in recent years [45]. Basically, studies of biodegradation are focused on polymer composites containing a filler, which has an accelerating effect on biodegradation, being a source of nutrition for microorganisms in the external environment [46,47,48].

Despite the fact that polyvinyl chloride is a strong polymer, resistant to abrasion and chemical action, and characterized by low moisture absorption, researchers note that the lower the molecular weight of the polymer, the more easily its biodegradation occurs [49,50]. According to Kirbas et al., low molecular weight pure PVC is biodegradable by white rot fungi [51].

Research is also underway on the possibility of PVC biodegradation by fungi Aspergillus fumigatus [52]. For the same purpose, Phanerochaete chrysosporium was grown on PVC in agar with mineral salt [22,53,54]. It has also been shown that Phanerochaete chrysosporium, Lentinus tigrinus, Aspergillus niger, and Aspergillus sydowii can efficiently degrade PVC [55,56]. Thus, certain types of microorganisms are able to utilize polyvinyl chloride, and the additives present in composite materials significantly accelerate this process.

Environmental damage from pollution and deterioration of land resources under the influence of anthropogenic loads is expressed in soil degradation. The assessment of the value of the prevented environmental damage from soil degradation when using the developed additives was calculated by the Formula (1) [57]:

(1) $${У}_{{\mathrm{pr}}_{a\;}}^{s}={\;У}_{{\mathrm{sp}}_{х\;}}^{s}\times \underset{j}{\sum }S_{j\;}\times {\;K}_{\mathrm{nj}}\;,$$

$${У}_{{\mathrm{pr}}_{a\;}}^{s}=147000\times 1\times 1.9=279.3\;\mathrm{thousand}\;\mathrm{rubles}\;$$

where ${У}_{{\mathrm{sp}}_{х\;}}^{s}$—indicator of specific environmental damage to land resources, thousand rubles; ${У}_{{\mathrm{sp}}_{х\;}}^{s}$ = 147 thousand rubles/ha (Ufa c., Russia);

Sj—area of land of type j, preserved from degradation as a result of environmental protection, ha; Sj = 1 ha; 

Knj—coefficient of natural and economic significance of land resources of the j-type, Knj = 1.9.

The amount of prevented environmental damage as a result of environmental protection from the pollution of land resources by chemical substances is estimated by the formula (2):

(2) $${У}_{{\mathrm{pr}}_{х\;}}^{s}={\;У}_{{\mathrm{sp}}_{х\;}}^{s}\times \underset{j}{\sum }S_{j\;}\times {\;K}_{i}^{o}\times {\;K}_{\mathrm{nj}}$$

$${У}_{{\mathrm{pr}}_{х\;}}^{s}=147000\times 1\times 3\times 1.9=837.9\;\mathrm{thousand}\;\mathrm{rubles}\;$$

where ${У}_{{\mathrm{pr}}_{х\;}}^{s}$—prevented environmental damage from pollution of land resources with a chemical substance of the i-th hazard class during the reporting period, thousand rubles;

Sj—area of land of type j that was prevented from contamination by a chemical substance of the i-th hazard class during the reporting period of time, ha; Sj = 1 ha;

Ki^o^—coefficient taking into account the hazard class of the i-th chemical that is not allowed to enter the soil or eliminated pollution as a result of the implementation of the corresponding direction of environmental protection, Ki^o^ = 3.

The total amount of prevented environmental damage to land resources in all areas of environmental protection in the region during the reporting period is determined by summing up all types of prevented environmental damage by the formula (3):

(3) $${У}_{{\mathrm{pr}}_{\;}}^{s}={У}_{{\mathrm{pr}}_{a\;}}^{s}+{У}_{{\mathrm{pr}}_{х\;}}^{s}$$

$${У}_{{\mathrm{pr}}_{\;}}^{s}=279.3+837.9=1117.2\;\mathrm{thousand}\;\mathrm{rubles}.\;$$

Considering that the biodegradation of a polymer material takes more than 100 years, the environmental damage inflicted over a given period of time will amount to 1,117,200 rubles.

Since the obtained composite PVC–plastic compound with the content of adipate plasticizers degrades in the soil in 0.5 years to 10%, the complete biodegradation will be 5 years [58,59,60]. The environmental damage caused over a given period of time will be 55,860 rubles.

Thus, the prevented environmental damage during the incorporation of biodegradable adipate plasticizers into production for 100 years will amount to 1,061.34 thousand rubles per 1 ha of land.

## 4. Conclusions

New environmentally friendly plasticizers—asymmetric esters of adipic acid—have been obtained. The conducted thermoanalytical studies have shown that the developed additives are characterized by high thermal stability, good manufacturability, and a significant decrease in the glass transition temperature of polyvinyl chloride. The mycological test of samples of PVC films containing the obtained adipates and the study of changes in physical and mechanical characteristics confirmed the possibility of their biodegradation in natural conditions. An ecological and economic assessment of the use of new adipate plasticizers in PVC composites shows the effectiveness of their use to accelerate biodegradation processes. 

## 5. Patents

Patent 2,716,691 RU, Int. Cl. ^51^ C08L 27/00 (2006.01). Plasticizer for compositions based on polyvinyl chloride. Mazitova A.K., Aminova G.K., Vikhareva I.N., Sukhareva I.A., Zaripov I.I., Akhmetov I.R. Proprietor Federalnoe gosudarstvennoe byudzhetnoe obrazovatelnoe uchrezhdenie vysshego obrazovaniya “Ufimskij gosudarstvennyj neftyanoj tekhnicheskij universitet” (RU)—No. 2019131313; Date of filing 02.10.2019; Date of publication 13.03.2020.

## Figures and Tables

**Table 1:** Physicochemical properties of unsymmetrical esters of adipic acid.

Ester	Indicators			
	Molecular Weight	Acid Number, mg KOH/g	Ester Number, mg KOH/g	d^20^_4_
Decyl butoxyethyl adipate (DBEA)	388	0.1	288	1.1003
Decyl phenoxyethyl adipate (DPEA)	406	0.1	273	0.9957

**Table 2:** The results of the thermal analysis of the plasticizers.

Sample	Characteristics			
	Temperature, °C			Δm, %
	Beginning	Maximal Value	End	at 180 °C
DBEA	153	278	498	0.8
DPEA	101	316	499	0.9
DOP	132	284	497	1.0

**Table 3:** DSC analysis of the plasticizers.

Sample	Endotherm Characteristics		Exotherm Characteristics	
	Temperature, °C	ΔH_m_, J/g	Temperature, °C	ΔH_cr_, J/g
DBEA	−40.3	−14.1	−60.5	9.4
DPEA	84.0	−67.8	8.0	131.2

**Table 4:** The results of determining the critical temperature of the dissolution of the plasticizers.

Plasticizer	Sample Number	Tc, °C
DOP		113
DBEA	1	135
DPEA	2	169

**Table 5:** Evaluation of the biostability of PVC composites in points.

Composition Number	Micromycete		
	*Aspergillus niger*	*Pénicillium funiculosum*	*Trichoderma* *lignorum*
1	4	4	4
2	3	3	3

**Table 6:** Physical and mechanical characteristics of the obtained PVC compositions.

Composition Number	Tensile Breaking Stress, MPa	Elongation at Break, %
1	16,5	227
2	17,0	221

**Table 7:** Changes in the physical and mechanical characteristics of PVC compositions.

Exposure Time, Days	Composition 1		Composition 2	
	Change in Tensile Stress, %	Change in Elongation at Break, %	Change in Tensile Stress, %	Change in Elongation at Break, %
10	−7.1	1.5	−4.2	1.0
30	−21.4	3.4	−18.9	2.7
45	−43.3	7.4	−37.4	6.8
90	−64.9	15.7	−60.3	14.9
120	−95.7	31.2	−91.3	29.8

## Data Availability

The data presented in this study are available on request from the corresponding author.
